# Low Transmission to Elimination: Rural Development as a Key Determinant of the End-Game Dynamics of *Schistosoma japonicum* in China

**DOI:** 10.3390/tropicalmed2030035

**Published:** 2017-08-04

**Authors:** Robert Spear, Bo Zhong, Song Liang

**Affiliations:** 1 School of Public Health, University of California, Berkeley, CA 94720-7360, USA; 2 Institute of Parasitic Diseases, Sichuan Center for Disease Control and Prevention, Chengdu 610041, China; zhongbo1968@163.com; 3 Department of Environmental and Global Health, College of Public Health and Health Professions, and Emerging Pathogens Institute, University of Florida, Gainesville, FL 32610-3010, USA; songliang@ufl.edu

**Keywords:** *S. japonicum*, rural development, low transmission, elimination

## Abstract

Rural development has been a critical component of China’s economic miracle since the start of economic reform in the early 1980s, both benefiting from and contributing to the nation’s rapid economic growth. This development has yielded substantial improvements of public health relevance, including contributing to major reductions in schistosomiasis prevalence. The history of schistosomiasis elimination in Japan suggests that development played a dominant causal role in that nation. We argue that it is highly probable that a similar story is playing out in at least some large regions of China. In particular, we summarize evidence from Sichuan Province which supports the case that economic development has led to improvements in rural irrigation and water supply which, together with changes in crop selection and agricultural mechanization, have all contributed to sustainable reductions in the prevalence of *Schistosoma japonicum*. The two major factors that have experienced major reductions are the area of snail habitat and the degree of human exposure, both through a variety of mechanisms which differ by region and economic circumstance. However, hotspots of transmission remain. Overall, however, economic development in traditionally endemic areas has provided the resources to carry out projects that have had major beneficial impacts on disease transmission that are likely to be sustainable.

## 1. Background

Rural development has been a critical component of China’s economic miracle since the start of economic reform in the early 1980s, both benefiting from and contributing to the nation’s rapid economic growth. This development has yielded substantial improvements that are of public health relevance including, but not limited to, income and living conditions (e.g., sanitation facilities), infrastructure (e.g., centralized drinking water systems), roads and transportation networks, services (e.g., access to health care), agriculture (e.g., mechanization and improved irrigation), and increasing countryside urbanization (e.g., integration of urban and rural areas) [1]. These developments have dramatically transformed rural landscapes throughout the country. In Sichuan Province, for example, from 1985 to 2015, the annual per capita farmer’s income has increased almost 33-fold, from $46 to approximately $1570, energy utilization for agricultural mechanization has increased 6-fold, from 7 million kilowatts to 41.6 million kilowatts, and access to centralized piped water has increased from about 5% to close to 60% among rural communities during the same period [2].

Yet, development has shown substantial regional disparities, in part due to differences in the underlying economies because the financing of development usually comes from all three levels of governmental administration—central, provincial, and local [1]. For example, based on a 2006–2007 national survey, the coverage of improved sanitation and water quality reached 100% and 88% respectively in rural areas of Shanghai, whereas in Sichuan, these coverages were 45% and 33%, clearly indicating the wealthy-poor divide [3,4]. Similar differences occur within regions as well. In Sichuan during the same period, for example, the coverage of improved sanitation and water access were more than 80% and 76%, respectively in the Chengdu plains, whereas in the Xichang mountainous area these coverages were 38% and 44% [5].

The public health sector has taken advantage of development in its various aspects, integrating it into schistosomiasis control programs, for example, that are the focus of this report. Viewed first from a national perspective, the longstanding and persistent efforts of the central government to suppress the transmission of *S. japonicum* in China have met with considerable success [6,7]. In 1958 when the first national survey was completed it was estimated that 10 million were infected and over 100 million were at risk [7]. By 2004 the number infected had been reduced to below 850,000 and in 2013 the estimated number had further decreased to 185,000 with only 9 of these reported cases being of acute disease. Prior to the availability of the chemotherapeutic drug praziquantel in the 1980s, the principal control strategy responsible for this success relied on suppressing populations of *Oncomelania* snails, the intermediate host of the parasite. Subsequently, a combination of snail control and the use of praziquantel was widely and effectively used which remains the case today in refractory or re-emergent areas.

In the last decade a comprehensive strategy was implemented that involves a much broader set of interventions mainly focused on environmental determinants of transmission and on bovine populations. During this period, however, disease transmission has periodically re-emerged in a number of settings where control had been achieved [8]. It is not clear whether this has been due to import from other areas, previously undetected local disease in humans and/or transmission from animal reservoirs. A further complication is that it appears that low-level transmission can continue below the sensitivity of traditional surveillance methods [9], although new and highly sensitive methods have been reported [10]. Regardless of the cause, the current challenge is to identify areas in which transmission continues, albeit in isolated hotspots and at quite low levels, and move those sites from low transmission to elimination.

Our work in irrigated agricultural settings in Sichuan Province has focused on the environmental determinants of transmission and has underscored the need for integrated controls to sustainably reduce the basic reproductive number, *R*_0_, to below unity, the threshold below which disease transmission should continually diminish to zero without further interventions [11,12]. However, even in settings where the effective reproductive number is near or below unity, theory predicts, and some epidemiological evidence supports, that the rate of change in the prevalence and intensity of human disease in the absence of environmental change is often very slow and elimination may be achieved only after a decade or more [13]. This general effect has been observed with other environmentally-mediated infectious diseases, an example being lymphatic filariasis [14]. The successful history of reducing schistosomiasis in China and the strategies used to achieve these reductions suggests that environmental change is likely to have played a significant role, particularly in recent years. This is in contrast to the history of dramatic increases in transmission resulting from water development projects, the classic example being the construction of the Diama Dam on the Senegal River in Africa. However, as noted above, rural development is now occurring in much of China at a pace that suggests that environmental change in many areas historically endemic for *S. japonicum* are likely to be experiencing greater or more sustainable reductions in transmission, and over shorter time spans, than control programs, theory, or earlier epidemiological data would predict. The history of the elimination of *S. japonicum* transmission in Japan suggests that both agricultural development, and rural development more generally, played a dominant role in their success. We begin with a brief review of the Japanese experience and then turn to the recent experience in Sichuan with the objective of better understanding how best to take advantage of rural development to hasten progress to disease elimination.

## 2. *S. japonicum* Elimination in Japan

A 1996 paper by Tanaka and Tsuji presents an excellent review of the Japanese experience from 1847, when the symptoms of schistosomiasis infection in humans were first reported, to after the last known human cases in 1977 [15]. Notably, the last human case in Japan was reported several years before the introduction of praziquantel, the world-wide use of which continues to have major impact on reducing the morbidity arising from chronic or repeated schistosomiasis infections. In the 1930s antimonyl potassium tartrate, Stibinal, was the drug of choice for treating schistosomiasis in Japan, but it required daily injections for almost 3 weeks, caused severe side effects and did not reliably result in complete deworming [16]. As a consequence, the literature implies that Stibinal and other drugs were used to treat advanced cases and that there was no analog, common in China and other endemic regions in recent years, to the use of praziquatel in mass drug administration (MDA) programs in which all exposed individuals in an endemic area are treated. Hence, control efforts in Japan focused on direct snail control and environmental modifications resulting in the destruction of snail habitat.

In Japan there were three major endemic areas and three minor areas. The environmental conditions in all were initially well-hydrated and hospitable to the intermediate host of the parasite, *Oncomelania nosophora* snails. Rice-growing areas, marshlands, and river banks were typical snail habitat. By 1920 the role of the snail as an intermediate host of the parasite was established, and control efforts aimed at reducing snail populations had begun. Various means of direct snail control were used over the ensuing years including boiling water, flame throwers, and various chemical mollusicides. Environmental changes that sustainably diminished snail habitat became more common in later years when drainage and water management improvements, including concreting of irrigation ditches and crop changes from rice paddies to orchards all had the effect of reducing snail habitat. The situation was summarized in 1965 by Yokogawa [17] in addressing the status of the disease in the Kofu Basin of Yamanashi Prefecture which he characterized as the most important of the endemic areas in Japan at that time:

“…..many of the breeding places for vector snails have been removed and cases of schistosomiasis have decreased rapidly to a point where acute cases of schistosomiasis are very rare now. This is no doubt due in part to thorough spreading of molluscicides and the construction of concrete ditches. But certainly major factors have been the decrease in farming area due to urbanization, transformation of farmland into land for building, and conversion of rice fields into orchards or mechanized farms. In 1956 the area in which *Oncomelania nosophora* was found extended to 18,000 hectares with a farm population of 370,000; by 1966 the breeding area had been reduced to 7333 hectares and the population of farm laborers in danger of infection had dropped to 42,751.”

To understand how development and these various interventions impacted the prevalence and intensity of the disease in both the short and long-term, it is necessary to look more closely at the structure of the schistosomiasis transmission cycle. As mentioned above, by 1920 the basic structure of the transmission of *S. japonicum* was understood; the adult worms reside in the vasculature surrounding the liver of the definitive host; each mated female produces copious amounts of eggs, some of which are excreted in the feces of the definitive host and find their way into surface waters where they hatch into free-swimming miracidia. This form of the parasite then seeks an amphibious snail of the species *Oncomelania* to infect. Successful infection and maturation of the parasite in the snail leads, in turn, to the release of a second free-swimming form, cercariae, which then seek a mammalian host to infect and complete the cycle. The progression of the pathology of the disease depends on the number of worms harbored by the host, referred to as the worm burden, which also controls the number of eggs excreted into the environment. In China the dispersion of eggs into the environment continues to be facilitated by the use of mixed human and animal feces as fertilizer.

## 3. Methods

While the causal links between the various elements of the transmission cycle have been long understood, their interaction and impact on the intensity of infection in humans or snails requires a more quantitative description. Here we draw on a substantial history of the use of mathematical models to study various aspects of the time course of the transmission cycle as well as the effects of control strategies, most often mass drug administration (MDA). We have also used models in our studies of the environmental determinants of human exposure and its control. Here we use a community model as a means of describing the mechanisms by which disease transmission is altered by both historical and current control strategies and to explore the impact of rural development in moving from low transmission to elimination. The term community model refers to a differential equation model that tracks the mean worm burden in a human population and the mean density of infected snails residing in their surface water environment. While an individually-based model is more appropriate for quantitative studies at low transmission intensities or to assess stochastic effects, the causal linkages and main effects are the same in both.

The most common community model of schistosomiasis transmission is based simply on the concept that the rate of change of mean worm burden in the human population is the difference between the rate at which worms are acquired and mature into adults in vivo and their death rate. Similarly, the rate of change of infected snail density is the difference between the rate of infection of snails by schistosome miracidia and the death rate of these infected snails. A version of such a model that we have used extensively is treated in detail in [12]. Even this structurally simple model can serve as a platform to discuss control strategies because it incorporates a variety of determinants of transmission intensity. These include density-dependent constraints on worm establishment in the host, time-variable parameters that relate to uninfected snail density, several temperature-dependent processes, and seasonal water contact of the human population.

Here we cite this model because it allowed the derivation of an explicit expression that includes the important site-specific determinants of the basic reproductive number. Recall that the basic reproductive number, *R*_0_, is defined for schistosomiasis as the number of female worms reproduced by one female worm during its lifetime in the absence of acquired immunity in the host population. In common with other infectious diseases, if $R_{o}<1$ transmission will approach zero over time from any non-zero initial state. Conversely, if $R_{o}>1$ , transmission will persist and reach some endemic level over time. However, because the model used here as the foundation of our qualitative analysis includes time variable parameters and acquired immunity, an approximation to *R*_0_ is used in which the time variable parameters are averaged over the annual cycle to derive an effective reproductive number, $R_{eff}=P_{b}P_{s}$, where $P_{b}$ depends on the relevant biological properties of the parasite, the snail, and the human host that should be at least regionally invariant. The second, the site-specific parameters, $P_{s}$, summarize the effects of environmental determinants of transmission intensity and, more importantly for present purposes, the effects of interventions aimed specifically at diminishing the intensity of disease transmission because of planned or unplanned effects of rural development. Specifically:

$$P_{s}=\frac{S_{m}\gamma A_{h}X_{m}\beta \xi ng_{0}{\bar{\alpha }}_{12}{\bar{\alpha }}_{21}}{A_{s}^{2}}$$

where *S_m_* and *γ* relate to human water contact, $X_{m}$ to uninfected snail density, $\beta ng_{0}$ to the intensity of parasite-contaminated fertilizer use and $A_{h}$ and $A_{s}$ the area of snail habitat and surface water in the village, respectively [11]. Both ${\bar{\alpha }}_{12}$ and ${\bar{\alpha }}_{21}$ vary between zero and one and account for seasonal factors, notably variations in human water contact, uninfected snail density, and the annual temperature and rainfall profiles.

A final point of both practical and methodological importance is that interventions to diminish transmission are of two sorts. One type is aimed at reducing the values of the site-specific parameters comprising *P_s_* and the other directly reduces the population of parasites in the human host or the population of snails, both infected and uninfected. This second type of intervention targets what are termed the state variables of the model rather than the parameters controlling the rates of change of these populations. However, only interventions that alter the parameters, in the present context specifically *P_s_*, change *R_eff_*. For example, well-conducted MDA programs can reduce village worm burden by about 95%, but without altering *R_eff_* or the underlying *R*_0_. It is possible, however, for MDA to terminate transmission, but if *R*_0_ > 1, the parasite can be easily reintroduced and the worm burden will begin to rebound towards its endemic level. Similar phenomena, but unrelated to infectious diseases, occur in ecology more generally [18].

**Figure 1:**
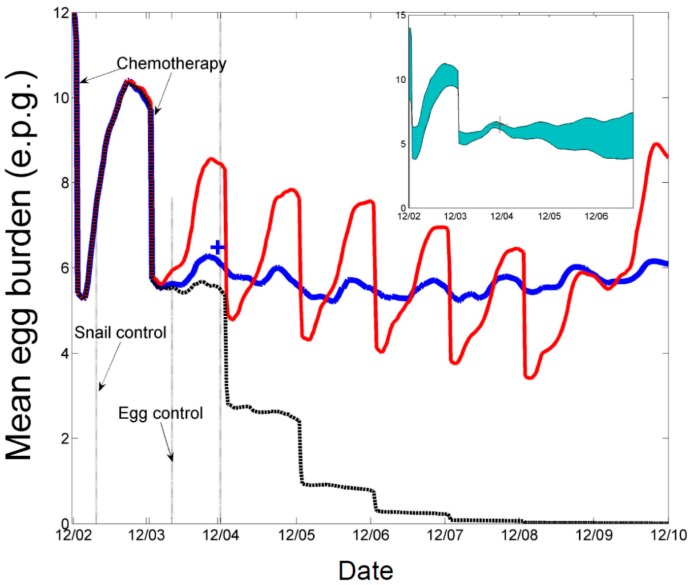
Time profiles of median infection intensity for farmers in Chuanxing Xinlong 7 under three hypothetical control scenarios. Scenario I, **blue**, two chemotherapies in 2002 and 2003, coupled with sustained environmental interventions to 2010; II, **red**, chemotherapies as in scenario I followed by annual chemotherapy at 50% coverage to 2008, no environmental interventions; III, **black**, a combination of both scenarios I and II. The inset in the upper right of the figure shows the variability model predicts around the blue line due to residual uncertainty in parametric values.

An example of the effects of the two types of interventions is shown in Figure 1 [11], from an earlier study. It shows the median infection intensity, measured in schistosome eggs per gram of feces as determined by the Kato-Katz procedure, as simulated by a version of the community model that includes time varying parameters, among farmers as a result of several types of hypothetical interventions in a village in Sichuan Province. The red line shows the effects of annual mass chemotherapy from 2002 to 2008; the blue line shows the effects of the 2002–2003 chemotherapies followed by a 50% reduction in *P_s_*; and the black line annual chemotherapies until 2008 plus the 50% reduction in *P_s_*. While the effective reproductive number, *R_eff_*_,_ has been reduced by 50% in this hypothetical example, it remains above unity as shown by both the red and blue lines, indicating that re-emergence is possible even after sustained chemotherapies to 2008 if the parasite is reintroduced to the village after that time. The point, however, is that sustainable reductions in *P_s_* are necessary to achieve long-term reductions in infection intensity, but chemotherapy of humans and molluscicide use can hasten the time required to reach this reduced level.

## 4. Results

We now return to the Japanese experience with the objective of interpreting that history in the context of its effects on *R*_0_ via changes in *P_s_*. Recall that transmission of *S. japonicum* in Japan had largely been terminated prior to the advent of praziquantel and the literature does not suggest that earlier drugs were used except for strictly medical treatment of advanced cases. Hence, the snail control interventions and/or development-related effects affect five elements of *P_s_* which together control the exposure intensity of cercariae and miricidia to which the human and snail populations are exposed. These are the maximum annual density of infected snails, *X_m_*, the area of snail habitat, *A_h_*, the area of surface water into which cercariae and miracidia are distributed, *A_s_*, the population of individuals exposed, *n* and *S_m_*, the maximum annual water contact level of the human population. The parameter *A_s_* will not be regarded as an independent intervention target because snail habitat is closely related to surface water hydrology causing a high correlation between the area of snail habitat *A_h_* and *A_s_.* An exception is the concreting of ditches which reduces *A_h_* but leaves *A_s_* unchanged. In addition, the two *α* parameters, which account for the time variation of snail density and water contact, are included for technical completeness.

Table 1 summarizes the relation between environmental changes noted in the Japanese literature and the elements of *P_s_* thereby affected. Clearly, most of the interventions listed in the table sustainably reduce the area of snail habitat and the extent of human contact with surface waters in the local environment. Since *P_s_* depends on the product of *A_h_* and *S_m_*, an intervention like a change in crops from rice to tree crops, for example, that might reduce both by 50%, would lead to a 75% reduction in *P_s_*. In that case, a *R_eff_* less than 4 would be reduced to less than 1 leading to a continual decrease in infection intensity to the zero state, albeit over a number of years, with no further intervention. While values of *R_eff_* based on field data are rare, our work in Sichuan suggests that in 2000 the most intensive transmission we observed at the single village level was associated with *R_eff_* values in the range of 2.5–3.0, which would require decreases in *P_s_* of about 60–70% to achieve long-term elimination.

### 4.1. Rural Development and Schistosomiasis in Sichuan

**Figure 2:**
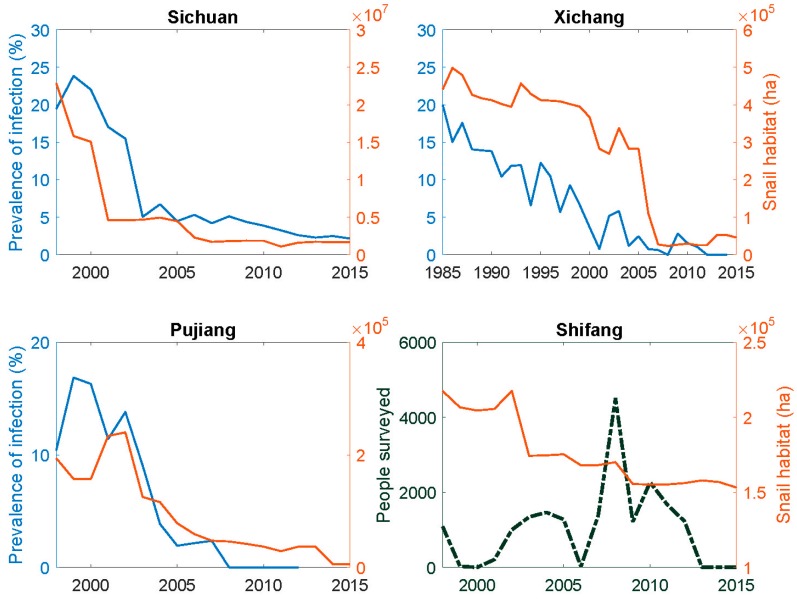
Historical trends of schistosomiasis prevalence of infection and intermediate snail host habitat for Sichuan Province (1998–2015), Xichang (1985–2015), Pujiang (1998–2015), and Shifang (1998–2015) counties. For Shifang county, the dotted line shows number of people surveyed each year, as only 40 people (out of 1088) were found infected in 1998.

In China, schistosomiasis transmission is classified as belonging to several different ecological classes. Sichuan falls into the hilly and mountainous class, which is largely comprised of irrigated agricultural land in which a variety of crops are cultivated including rice, tree crops, tobacco, wheat, vegetables, and rapeseed. While *S. japonicum* can infect 40 or more mammalian species, humans and bovines are regarded as the most important hosts. Figure 2 shows the aggregated human prevalence data for the 44 endemic counties of the province from 1998 to 2015 as well as the area of snail habitat based on annual reporting to Sichuan CDC from county-level surveys. The figure also includes plots of similar data from the three counties addressed in greater detail below. Note, however, that the province-wide prevalence data is based on immunoassay results in contrast to the county data that is based on analysis of fecal samples using either the Kato-Katz procedure or a miracidial hatch test widely used in China. We speculate that the province-wide data somewhat overstates the number of active infections, particularly at higher prevalence. Nevertheless, the dramatic decrease in both prevalence of human infection and the area of snail habitat converges to very low levels in all these data by 2015.

**Figure 3:**
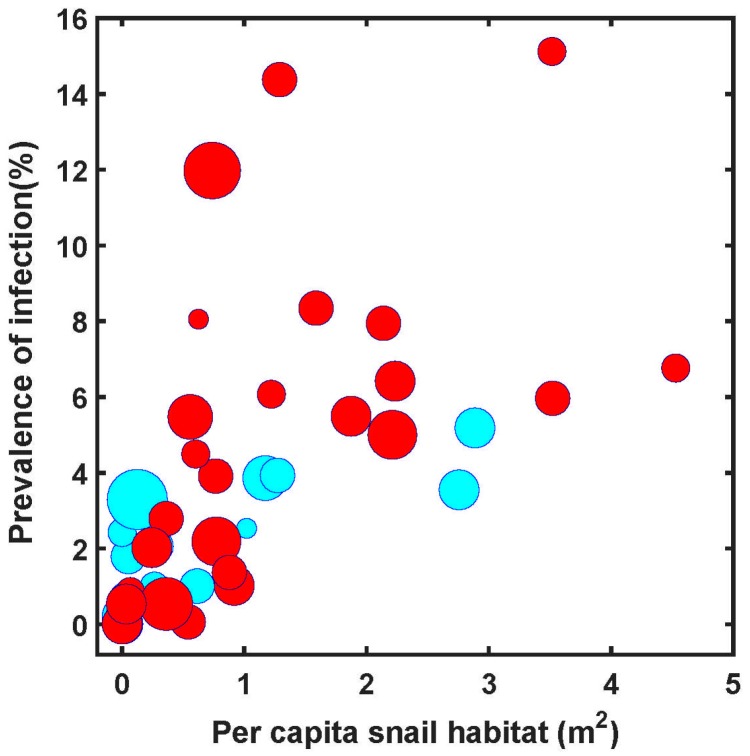
The relationship between prevalence of schistosomiasis infection and snail habitat for 44 endemic counties in Sichuan based on 2008 data, showing a significant correlation (R^2^ = 0.371, *p* < 0.01). The 44 endemic counties included 18 which achieved transmission control (**cyan**) and 26 with ongoing transmission (**red**) in 2008. The sizes of circles indicate relative magnitude of per capita GDP by county.

**Figure 4:**
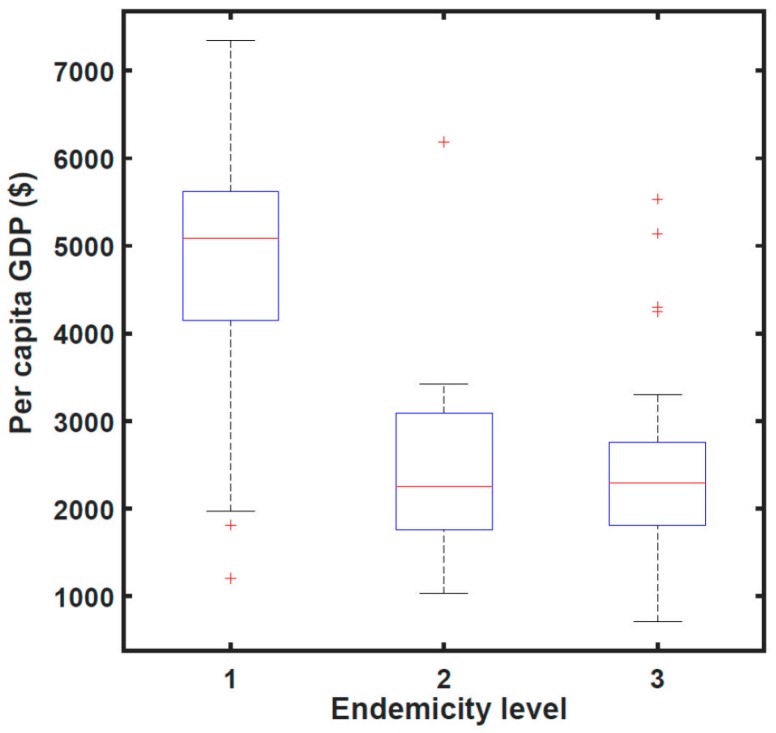
The relationship between endemic level and per capita GDP by county in 2008. The three levels of endemicity are, 1—transmission interruption (18 counties); 2—transmission control (18 counties); and 3—active transmission (26 counties). Overall, counties in level 1 had significantly higher per capita GDP (average $4291, *p* < 0.01) than that for level 2 counties ($2252) and level 3 counties ($2201).

Figure 3 shows the relationship between human prevalence and snail habitat per person at risk for the same 44 endemic counties in 2008, distinguishing between the 18 counties where transmission had been controlled and the remaining 26 where active transmission continued. These data suggest that prevalence decreases with per capita snail habitat but other factors are clearly involved. Figure 4 shows data, again for 2008, that wealthier counties were more likely to have achieved transmission elimination than poorer counties.

The dramatic decline in human prevalence in recent years and the lack of sensitivity of traditional methods of disease surveillance make recent province-wide estimates somewhat unreliable. Clearly hotspots of transmission remain, but their detection is challenging. Further, and not surprisingly, at the provincial level of data aggregation it is difficult to gain much insight into just how reductions in snail habitat were achieved or how increased wealth was utilized to lead to decreases in the prevalence of disease. Hence, we have chosen three counties with different agricultural and economic circumstances to look more closely at their route to disease suppression.

### 4.2. Xichang County

Endemic areas in Xichang County represent the typical mountainous transmission environment. The region is characterized by a less-developed economy in comparison with those in the plains and hilly region of the province. Among 26 endemic counties with active transmission in 2008, nine were from mountainous regions accounting for 75% of the mountainous endemic counties. In this type of transmission environment, human prevalence of infection was historically high. Many factors contributed to this, primarily a lack of improved water sources, sanitation, and hygiene (WASH), as well as the wide distribution of intermediate snail hosts along crisscross irrigation ditches in terraced fields where typical environmental management approaches (e.g., concrete-lined ditches) are challenging to implement. In addition, extensive human exposure due to the nature of agricultural activities was common. Our previous study in Chuanxing and Daxing townships (about 5 × 5 km areas) in 2001 indicated that human prevalence of infection at the village level ranged from 2 to 55% [19]. Typical control programs relied principally on drug treatment and mollusiciciding until the early 2000s, when an agriculturally-oriented comprehensive program was launched. The program largely focused on conversion of slopes to terraces, wetland to dryland (e.g., via drainage systems), and alteration of crop structure (e.g., to less water-demanding crops). In suitable and limited areas, irrigation ditch lining with concrete and household-based sanitary toilets, generally involving biogas digester installations, were also implemented. As of 2015, about 40% of wetland in endemic villages had been drained and 30% of irrigation ditches were lined with concrete. Such programs have reduced snail habitat by 90%, from 440,892 ha in 1985 to 44,488 ha in 2015, while human prevalence of infection decreased from 20 to 0.56% in the same period (Figure 2).

### 4.3. Pujiang County

Pujiang County, in a typical hilly region of the province, is located in the southwest corner of Chengdu Plains. Historically, the endemic level of schistosomiasis was among the highest in the hilly endemic areas. The average human prevalence of infection in endemic villages was around 25% in 1985. The main factors contributing to transmission included water development projects (e.g., reservoirs for irrigation) and relatively large populations of cattle that were the main reservoir of infection. Comprehensive development programs were initiated by the hydrologic and agricultural authorities in late 1990s and early 2000s. Projects focused on:

- a massive agricultural structure conversion (e.g., from paddy rice to less water demanding crops such as fruit trees and tea),
- improving domestic water sources for both humans and domestic animals and installing micro-irrigation systems,
- relocating typically scattered household residences to centralized communities,
- establishing agricultural corporations with more centralized and managed resources.

By taking advantage of such developments, public health professionals have worked with the hydrological and agricultural agencies to target high risk environments. As a result, snail habitat was reduced by 98%, from 193,872 ha in 1998 to 5328 ha in 2015 while no new human cases have been found since 2008 (Figure 2).

### 4.4. Shifang County

Shifang, located in Chuanbei area, represents a plains type of transmission environment. In the 70s, human prevalence of infection in the endemic villages ranged from 12 to 56%. Because of the more advanced economy in Shifang, in comparison with Xichang and Pujiang counties, the development program has focused on WASH improvement, mechanized agriculture, and development of a rural courtyard economy (e.g., household-based workshops). As of 2015, the coverage of sanitary toilets, mostly involving biogas digesters, reached more than 65% of households in endemic villages. The county reached transmission control in 1986 and experienced no new human infections since 1999 despite the existence of a relatively large remaining area of snail habitat.

**Figure 5:**
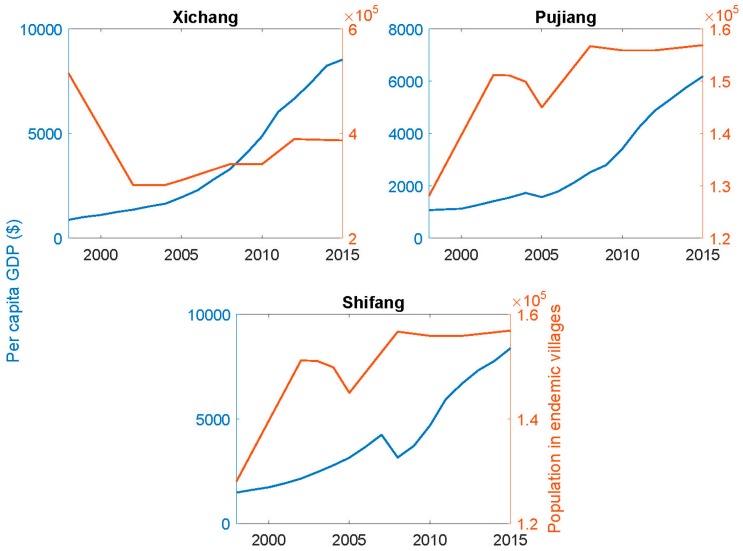
Per capita GDP and resident population numbers in endemic villages in the three counties.

It is clear from Figure 2 that the dramatic decrease in the area of snail habitat alone is sufficient to have sustainably reduced the site-specific determinants, *P_s_*, of the effective reproductive number for schistosomiasis transmission to near zero in Pujiang county if, as the data suggests, there is no remaining snail habitat and, in particular, no unidentified pockets of *Oncomelania* snails. A similar reduction in *P_s_* is also the case in Xichang and Shifang counties and throughout Sichuan on average, but some habitat remains and hotspots of transmission are not precluded. Table 2 summarizes the principal changes that produced these reductions in habitat as well as the other determinants of *P_s_*. Figure 5 shows the accompanying changes in the per capita GDP and in populations resident in the endemic villages. While there have not been dramatic changes in the population numbers over time, the GDP levels have increased markedly as noted above for Sichuan as a whole. However, there have been notable changes in the composition of the full-time resident populations in formerly endemic areas in that younger people, males in particular, often migrate to urban centers for employment.

## 5. Discussion

While we cannot extrapolate with quantitative confidence from Sichuan to other endemic areas of China, similar forces are at work. If the effects are also similar, the endgame is one of identifying and addressing the remaining hotspots of schistosomiasis transmission. This is, of course, easier said than done. Klepac et al. summarize the endgame challenges for infectious diseases in general [20]. In addition to the challenges posed by the insensitivity of traditional surveillance tools at low levels of transmission of *S. japonicum,* two disease-specific challenges relate to the involvement other mammalian hosts, notably bovines, and connectivity, the latter referring to social and physical interconnections between host populations of both snails and mammals. The role of cattle and water buffalo in schistosomiasis transmission has long been recognized and surveillance programs continue under the auspices of veterinary authorities. The issue of connectivity, which is of both practical and theoretical importance, suffers from a lack of field data to inform surveillance and control activities. While reasonable estimates can be made of the distance infected humans or bovines might travel beyond their home village, we know of only a single study of the distance cercariae can travel in an irrigation system and remain infective, that distance being greater than 400 m [21].

In much of our previous work and in the foregoing discussions, the effective reproductive number, *R_eff_*, pertained to a population living in a natural village and farming land adjacent to their residences. In an analysis of data from the 10 of the 20 villages we studied in Xichang County beginning in 2000, we found evidence that only two had *R_eff_* values consistent with internally sustained endemic infections and that infections in the other eight were likely to have been due to connectivity [12]. Gurarie and Seto [22] carried out an extensive theoretical study of a network of hydrologically and socially connected villages and concluded that “transmission can be sustained regionally throughout a group of connected villages even when individual village conditions appear not to support endemicity.” Hence, the notion of a hotspot of transmission may be a single village, but also it may be a more complex and less obvious connected group of intermediate and definitive hosts.

Some insight into the nature of hotspots remaining in Sichuan is offered by findings from a 2016 *S. japonicum* infection survey in suspected residual transmission hotspots, which indicated human infections in most of the 10 villages surveyed (E. Carlton, personal communication) [23]. None of these were of acute infections. These data were from hilly regions where we speculate that development has not yet reached the point at which the combination of human exposure and snail habitat reduction have resulted in sufficiently low values of *R_eff_*. Based on the distribution of infected humans and bovines, we suspect that these villages include both individual villages with *R_eff_* > 1, as exemplified in Figure 1, and transmission supported by low *R_eff_* values on networks of connected environments.

## 6. Conclusions

The reasons underlying the reductions in the prevalence of *S. japonicum* in the three counties in Sichuan province over the past 20 years have notable similarities with what we know of the Japanese experience. Rural development that resulted in reductions in snail habitat and diminished human exposure by changes in agricultural characteristics, land-use, and water supply or management are the common factors. We believe the same pattern underlies similar reductions in disease prevalence in the province as a whole and, very likely, in other endemic areas of China. With the exception of molluscicide application, none of these important changes in the rural environment summarized in Table 2 are under the direct control or budgetary allocations of public health agencies. Hence, communication and coordinated planning between governmental sectors is essential in making further progress. Certainly the longstanding national focus on the prevention of schistosomiasis has influenced rural development activities in recent years, but to what extent is difficult to estimate. Overall, however, economic development generally and in the three counties, in particular, has provided the resources to carry out development projects that have had major beneficial impacts on disease transmission that are likely to be sustainable.

The similarities between the situations we have described in Sichuan and in Japan suggest that economic development has been a major element of the suppression of *S. japonicum* transmission in those settings because it has supported rural improvements that lead to the sustainable local reductions in the basic reproductive number. A more rigorous analysis of the spatial characteristics of the temporal patterns we have described might offer more generalizable guidance in other environments or for other environmentally-mediated diseases.

We end on a note of caution in that there are other aspects of environmental change that are ongoing and may have less beneficial effects than those discussed above. The lake behind the Three Gorges Dam, for example, links two areas of the Yangtze watershed formerly endemic for schistosomiasis, has stabilized water levels in the Gorges and below the dam and is subject to silting, all of which raise the possibility of substantially increasing snail habitat [24,25]. Of even greater scale and uncertain effect is the prospect of climate change that is of concern for all environmentally-mediated infectious diseases. New challenges loom on the horizon.

## Figures and Tables

**Table 1:** Environmental improvements affecting determinants and effect duration of the site-specific elements of the effective reproductive number governing schistosomiasis transmission in Japan.

Environmental Change	*P_s_* Parameters	Effect Duration
Molluscicide applied	$X_{m},{\bar{\alpha }}_{21}$	Transient reduction
Concrete ditch	$A_{h}$	Sustained reduction
Drainage improvements	$A_{h},S_{m},{\bar{\alpha }}_{12}$	Sustained reduction
Crop change	$A_{h},S_{m},{\bar{\alpha }}_{12}$	Sustained reduction
Urbanization	$A_{h},n$	Sustained reduction
Agricultural mechanization	$S_{m},{\bar{\alpha }}_{12}$	Sustained reduction

**Table 2:** Environmental improvements affecting determinants of the site-specific elements of the effective reproductive number governing schistosomiasis transmission in three endemic counties of Sichuan province. + indicates significant level of improvement and ++ major extent of improvement.

Environmental Improvements	*P_s_* Parameters	Xichang	Pujiang	Shifang
Molluscicide applied	$X_{m},{\bar{\alpha }}_{21}$	+	+	+
Concrete ditch	$A_{h}$	+	+	
Drainage/irrigation improvements	$A_{h},S_{m},{\bar{\alpha }}_{12}$	+	++	
Crop/land use change	$A_{h},S_{m},{\bar{\alpha }}_{12}$	+	++	++
Community centralization	$\beta n,S_{m},{\bar{\alpha }}_{12}$		+	
Agricultural mechanization	$S_{m},{\bar{\alpha }}_{12}$			+
Domestic water supply	$S_{m},{\bar{\alpha }}_{12}$	+	+	+
Sanitary toilets/biogas	βn	+		++
